# A Brief Review on Fruit and Vegetable Extracts as Corrosion Inhibitors in Acidic Environments

**DOI:** 10.3390/molecules27092991

**Published:** 2022-05-06

**Authors:** Nnabuk Okon Eddy, Udo John Ibok, Rajni Garg, Rishav Garg, Amjad Iqbal, Muhammad Amin, Faisal Mustafa, Mehmet Egilmez, Ahmed M. Galal

**Affiliations:** 1 Department of Pure and Applied Chemistry, University of Nigeria, Nsukka 410001, Nigeria; okon.nnabuk@unn.edu.ng; 2 Department of Chemistry, Akwa Ibom State University, Ikot Akpaden 520221, Nigeria; udoibok@aksu.edu.ng; 3 Research & Development, Institute of Sci-Tech Affairs, Mohali 140306, India; rajnigarg@science.org.in; 4 Department of Civil Engineering, Galgotias College of Engineering and Technology, Greater Noida 201306, India; rishavgarg@science.org.in; 5 Department of Materials Technologies, Faculty of Materials Engineering, Silesian University of Technology, 44-100 Gliwice, Poland; 6 Department of Energy System Engineering, Seoul National University, Seoul 08826, Korea; amin7818@snu.ac.kr; 7 Department of Physics, American University of Sharjah, Sharjah 26666, United Arab Emirates; imfaisalmustafa@gmail.com; 8 Materials Science and Engineering Program, College of Arts and Sciences, American University of Sharjah, Sharjah 26666, United Arab Emirates; 9 Mechanical Engineering Department, College of Engineering, Prince Sattam Bin Abdulaziz University, Wadi ad-Dawasir 11991, Saudi Arabia; ahm.mohamed@psau.edu.sa; 10 Production Engineering and Mechanical Design Department, Faculty of Engineering, Mansoura University, Mansoura 35516, Egypt

**Keywords:** corrosion, green corrosion inhibitor, steel, phytochemicals, kinetics, adsorption

## Abstract

The corrosion of metals, i.e., the initiation and acceleration of the surface deterioration of metals through an electrochemical reaction with the surrounding intrusive environment, is a global concern because of the economic and environmental impacts. Corrosion inhibitors are considered the most practical choice among the available corrosion protection techniques due to their effectiveness in terms of functionality and cost. The use of traditional and toxic corrosion inhibitors has led to environmental issues, arousing the need for green counterparts that are environmentally friendly, easily accessible, biodegradable, and cost-effective. In this review, the utilization of green corrosion inhibitors purely acquired from renewable sources is explored, with an in-depth focus on the recent advancements in the use of fruit and vegetable extracts as green corrosion inhibitors. In particular, fruits and vegetables are natural sources of various phytochemicals that exhibit key potential in corrosion inhibition. To shed light on the true potential of such extracts in the protection of steel in acidic environments, the experimental techniques involved in corrosion inhibition and the mechanism of corrosion inhibition are discussed in detail. The study highlights the potential of fruit and vegetable extracts as non-toxic, economical, and effective corrosion inhibitors in the pursuit of green chemistry. In addition to discussing and outlining the current status and opportunities for employing fruit and vegetable extracts as corrosion inhibitors, the current review outlines the challenges involved in the utilization of such extracts in corrosion inhibition.

## 1. Introduction

**Figure 1:**
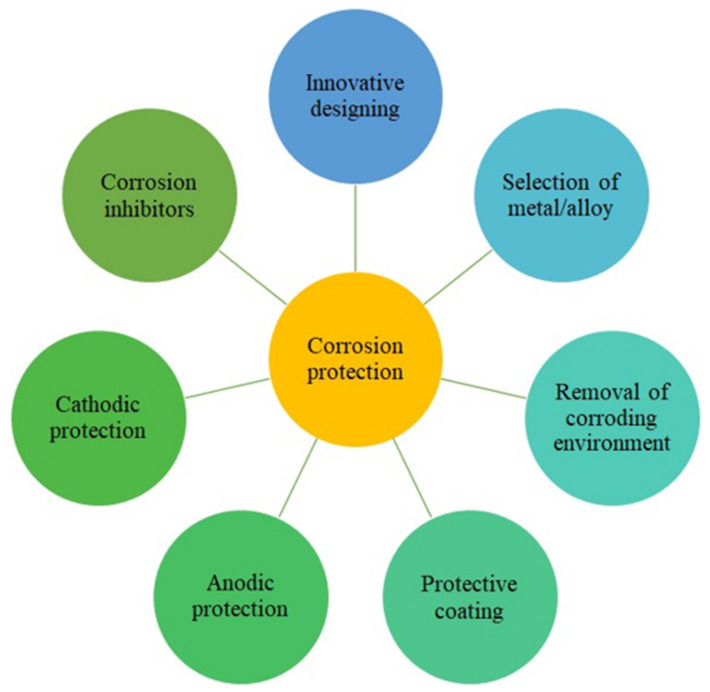
Widely utilized methods of corrosion protection.

The corrosion of metals is probably one of the most essential aspects of metallurgical sciences, as corrosion-related failures may result in disastrous health and environmental issues [1]. Coating, cathodic or anodic protection, electroplating, and several other methods illustrated in Figure 1 are useful approaches for the corrosion protection of metallic structures by isolating the surface from the corrosive environment [2]. As an alternative to the abovementioned methodologies, solution chemistry can be adopted to tune down the corrosion process at the metal–solution interface [3]. Such actions mostly include the use of corrosion inhibitors, which are additives that slow down the rate at which metal corrodes when added in minute quantities [4]. The action of a corrosion inhibitor can be altered or improved via an increase in the extent of adsorption of the inhibitor on the surface of the metal through the mechanism of physisorption or chemisorption or both [5]. An increase in the rate of diffusion of the inhibitor (in the bulk solution) to the surface of the metal or an increase in the miscibility of the inhibitor in the solution can be considered [6]. Favorable alterations in the anodic or cathodic reaction or both can also give the desired results [7,8]. Likewise, an increase in the resistance of the metal and a decrease in the corrosion current have also been reported as factors that can alter the mode of action of a corrosion inhibitor [9].

**Figure 2:**
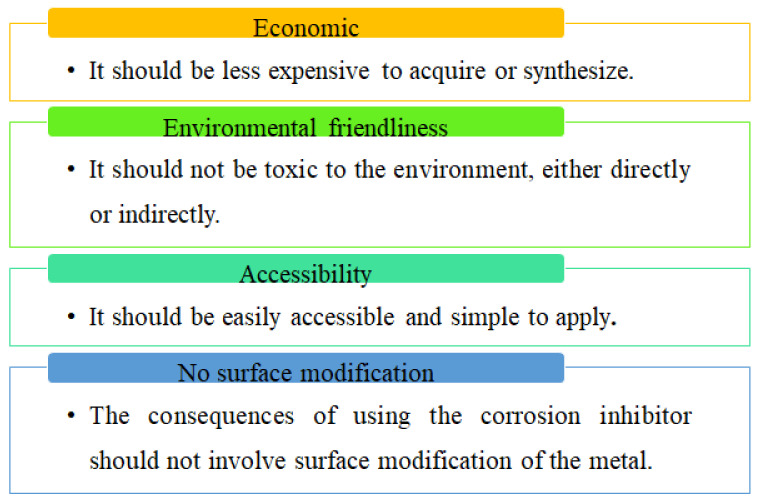
Characteristics expected from a good corrosion inhibitor.

The selection process of corrosion inhibitors has undergone several transformations over the years [4,5]. The expansion of research and development has given way to the development of a new set of corrosion inhibitors to meet the knowledge and functionality gaps created by the existing ones [10]. Such knowledge gaps that have been addressed in the research and selection processes come under the field of sustainability [11]. Years back, the development of corrosion inhibitors was focused on the use of inorganic salts or metal oxides, followed by a shift to organics, which has also received wider criticism in recent years due to the presence of unacceptable levels of heavy metals and other toxic compounds, poor solubility (especially in polar electrolytes), and reduced stability under critical conditions [12,13]. The corrosion inhibitors that have been in high demand in recent times are those that meet the conditions shown in Figure 2 [14]. In this respect, fruit and vegetable extracts serve as excellent candidates as non-toxic and sustainable corrosion inhibitors. Hence, the current study seeks to evaluate the current advancements, challenges, and opportunities in the field of corrosion inhibition with fruit and vegetable extracts.

Considering the above reasons, significant research effort has been spent on the use of organic corrosion inhibitors [4]. With the advent of green chemistry, the concept of green corrosion inhibition has aroused increasing attention [15]. A vast amount of literature exists on the use of natural products (amino acids, polyphenols, and alkaloids), ionic liquids, and plant extracts as green corrosion inhibitors [16]. The field of plant extract utilization in corrosion inhibition is gaining enormous acceptability and research interest, considering the increasing volume of research articles being published in the field of corrosion inhibition [17]. It is certain that there are several known and established corrosion inhibitors that have excellent inhibition efficiency compared to fruit and vegetables extracts, but the major limitation of these other classes of corrosion inhibitors (such as chromate, nitrite, etc.) is their failure to meet the environmental requirements [18]. This review is an overview of the work carried out on plant extracts as green corrosion inhibitors during the last decade. The basic techniques for the analysis of corrosion inhibition are explored. The challenges and future prospects for the widespread use of fruit and vegetable extracts as corrosion inhibitors are discussed.

## 2. Corrosion Inhibition Analysis

The corrosion of metal can be monitored using several quantitative approaches, including gravimetric, gasometric, thermometric, potentiodynamic, polarization, linear polarization, electrochemical impedance, and UV–visible spectroscopy techniques. The gravimetric method operates by monitoring the weight loss of the metal and is capable of providing information on the corrosion rate, inhibition efficiency, and degree of surface coverage [19]. The gasometric method monitors the volume of hydrogen gas evolved to obtain the inhibition efficiency [20]. The thermometric method employs the approach suggested by the thermodynamic explanation, namely that as the corrosion proceeds, the temperature of the medium is expected to rise. Therefore, the thermometric method monitors the changes in temperature as corrosion proceeds to obtain information for the reaction number (highest–lower temperature divided by time) and inhibition efficiency [21]. The potentiodynamic polarization method considers changes in corrosion current and the variations in electrode potential with the negative logarithm of the corrosion current, i.e., the application of the Tafel relationship [22]. In the linear polarization method, the response parameters are directed towards the variations in corrosion resistance with the potential, i.e., changes in polarization resistance [23]. The changes in charge transfer resistance as corrosion proceeds form the basis for the electrochemical impedance method of monitoring corrosion [24]. In corrosion studies, measurements of the wavelength of absorption and concentration of metal ions in the corrodents can be achieved via UV–visible spectroscopy [25]. The use of inductive coupling plasma–atomic absorption spectroscopy for the determination of the concentration of metal ions is also a commonly applied method [26]. The composition of the metal surface can be examined using X-ray fluorescence (XRF) spectroscopy, while X-ray diffraction spectroscopy (XRD) can be used to assess the crystal structure [27].

Existing non-quantitative methods include the use of Fourier transform infrared (FTIR) and scanning electron microscopy or total transmission microscopy (SEM/TEM) for the respective monitoring of the functional groups associated with inhibitors and their adsorption (i.e., FTIR) and for the examination of the surface morphology of the metal (i.e., SEM or TEM) [28].

Several theoretical physical–chemistry frameworks are useful in analyzing the effectiveness of a corrosion inhibitor, some of which include kinetic, thermodynamics, adsorption, and theoretical chemistry methods.

Kinetics studies are applicable for the formulation of the rate of corrosion, calculation of the activation energy, and prediction of the mechanism of adsorption. Most often, the kinetics of corrosion and corrosion inhibition is of the first order and obeys Equation (1):

(1) $$-\log\left(weight\;loss\right)=\frac{-k_{1}}{2.303}t$$

where $k_{1}$ is the rate constant and t is the period of immersion in hours or days. The activation energy for the adsorption process can be deduced from the Arrhenius equation (Equation (2)), which can be handled by plotting a graph of ln(CR) versus 1/T as Equation (2) or through a calculation that results in corrosion rates *CR*_1_ and *CR*_2_ at two different temperatures (*T*_1_ and *T*_2_, *T*_2_ > *T*_1_) as shown in Equation (3) [28]:

(2) $$ln\left(CR\right)=\ln\left(A\right)-\frac{E_{a}}{RT}$$

(3) $$ln\left(\frac{CR_{2}}{CR_{1}}\right)=\frac{E_{a}}{R}\;\left(\frac{1}{T_{1}}-\frac{1}{T_{2}}\right)$$

Thermodynamic theories estimate the exothermic or endothermic nature of the inhibition, calculate the orderliness of the adsorption process, and allow the estimation of the mechanism of adsorption [29]. Exothermic or endothermic adsorption depends on the change in enthalpy of adsorption ($\Delta H_{ads}$), while the degree of orderliness depends on the change in entropy of adsorption ($\Delta S_{ads}).$ Both parameters can be obtained from the Eyring transition state plot shown in Equation (4) [30]:

(4) $$ln\left(\frac{CR}{T}\right)=ln\;\left(\frac{R}{Nh}\right)+\left(\;\frac{\Delta S_{ads}}{R}\right)-\left(\frac{\Delta H_{ads}}{RT}\right)$$

where *R* is the gas constant, *T* is the temperature, *N* is Avogadro’s number, and *CR* is the corrosion rate of the metal. It has also been shown that the heat of adsorption can also be evaluated from the degree of surface coverage of the inhibitor at two different temperatures $\left(i.e.,\;\theta_{1}\;\mathrm{and}\;\theta_{2}\right)$ according to Equation (5) [31]:

(5) $$\Delta Q_{ads}=R\left[ln\left\{\left(\frac{\theta_{2}}{1-\theta_{2}}\right)-ln\left(\frac{\theta_{1}}{1-\theta_{1}}\right)\right\}\times \frac{T_{1}T_{2}}{T_{2}-T_{1}}\right]$$

Adsorption studies employ adsorption isotherm models to evaluate the adsorption characteristics of corrosion inhibitors and also to predict the spontaneity of the adsorption process (through the free energy of adsorption) [32]. There are various adsorption models, but the general behavior shows that adsorption models are rooted in the relationship between the concentration of the inhibitor in the bulk solution (C) and the degree of surface coverage of the inhibitor (θ). All adsorption isotherms have a constant term that is specific to such systems [33].

The theoretical chemistry approach allows the calculation of quantum chemical indices (i.e., molecular descriptors), evaluation of global and local reactivity parameters, and creation of links between the electronic parameters and inhibition efficiency and the proposed theoretical inhibition efficiency through quantitative structure–activity studies [34].

The increase in inhibition efficiency with concentration with respect to gravimetric, gasometric, and potentiodynamic polarization methods can be evaluated using Equations (6)–(8) [34,35,36]:

(6) $$\%IE=\left[\frac{CR_{b}-CR_{inh}}{CR_{b}}\right]\times \frac{100}{1}$$

(7) $$\%IE=\left[\frac{V_{Blank}-V_{Inhib}}{V_{Blank}}\right]\times \frac{100}{1}$$

(8) $$\%IE=\left[\frac{R_{Blank}-R_{Inhib}}{R_{Blank}}\right]\times \frac{100}{1}$$

where $CR_{b}\mathrm{and}\;CR_{inh}$ are the corrosion rates of mild steel when the inhibitor is not added and when it is added, respectively; $V_{Blank}\;\mathrm{and}\;V_{Inhib}$ are the volumes of hydrogen gas evolved by the solution without the inhibitor and in the presence of the inhibitor, respectively; $R_{Blank}\;\mathrm{and}\;R_{Inhib}$ are the reaction numbers for the blank solution and the acid solution containing the inhibitor.

## 3. Mechanism for Corrosion Inhibition

**Figure 3:**
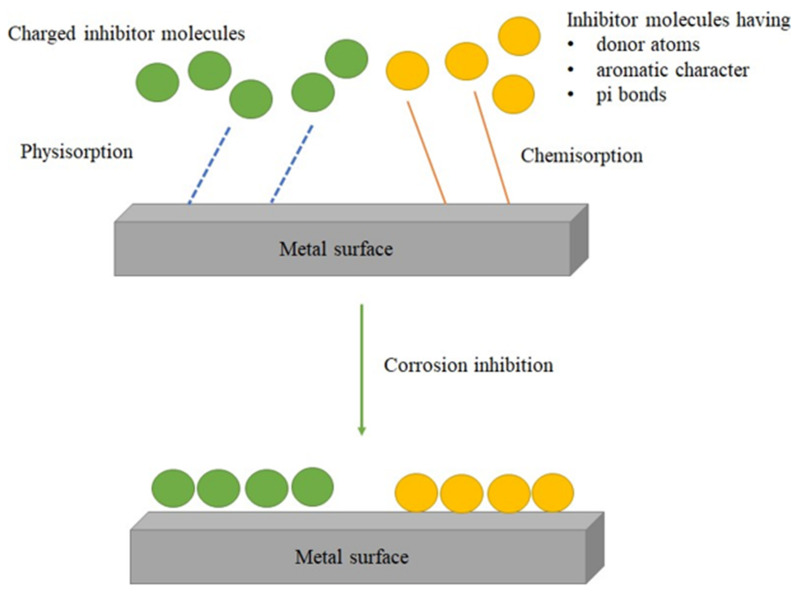
The mechanism for corrosion inhibition.

Corrosion inhibitors should meet sustainability requirements, which implies the tendency for such inhibitors to meet the present and future demands without the alteration of useful factors [35]. The inhibition action is considered to take place via physisorption or chemisorption. The weak polar interaction between the charged metal surface and inhibitor molecules results in physisorption, whereas in the case of chemisorption, the molecules are held onto the metal surface with strong electrostatic forces (Figure 3) [37].

It has been established that the adsorption of the inhibitor is spontaneous and due to the physisorption mechanism if the evaluated change in free energy ($\Delta G_{ads}^{0}$) is less than −40 kJ/mol., i.e., based on Equation (9) [38]:

(9) $$\Delta G_{ads}^{0}=-RTln\left(55.5K_{ad}\right)$$

where 55.5 is the molar heat of water and R is the gas constant.

In general, the extent of adsorption or the inhibition efficiency tends to decrease with increasing temperature in physisorption [39]. In the case of chemisorption, the electrostatic forces involve the interaction of non-bonding or anti-bonding electron pairs of metal inhibitors with activation energy. Hence, the inhibition efficiency tends to increase with increasing temperature. Efficient corrosion inhibitors meet some of the following conditions [40]:

1. Possession of heteroatoms;
2. Possession of suitable functional groups such as -NH_2_, OH, COOH, and multiple bonds;
3. Possession of aromaticity;
4. Possession of a long carbon chain and high molecular weight;
5. Solubility in aggressive solution.

The chemisorption behavior is further analyzed via computational studies, whereby the reactivity of the inhibitor can be predicted using frontier molecular orbital (FMO) theory. The sites for nucleophilic and electrophilic attacks are predicted using different Fukui function predictors. The binding energy between Fe and the inhibitor is computed as follows [41]:

(10) $$E_{Bind\;\left(inhibitor-Fe\right)}=E_{Bind\left(T\right)}-\left(E_{Bind\left(Fe\right)}+E_{Bind\left(Inhibitor\right)}\right)$$

where the subscripts $E_{Bind\left(T\right)}$ and $E_{Bind\left(Inhibitor\right)}$ are the total binding energy, the binding energy of iron, and the binding energy of the inhibitor, respectively.

## 4. Vegetable Extracts as Green Corrosion Inhibitors

Mild steel is one of the most commonly used materials in various industries, although it is likely to corrode under exposure to myriad conditions [42]. It reacts with non-oxidizing acids to form ferrous salts and an oxide layer is formed upon coming into contact with oxidizing acids. In either case, the steel surface is corroded along with exfoliation and dissolution of the metal (Fe) [4]. The corrosion inhibition of metals by various phytochemicals has been reported in the literature [37,43,44].

For example, Anupaama et al. [45] ascertained the role of phytochemicals in *Phyllanthus amarus* leaf for the inhibition of the corrosion of mild steel in HCl and was able to isolate certain phytochemicals that were significant in the corrosion inhibition process. Bhardwaj et al. [46] also identified certain phytochemicals as active ingredients for the inhibition of corrosion by most plant extracts and linked their corrosion inhibition potency to the structural properties of the phytochemicals, such as the possession of an aromatic structure, multiple bonds, and heteroatoms. A study conducted by Kumar and Mohana [47] also indicated phytochemicals in *Pterolobium hexapetalum* and *Celosia argentea* plant extracts as efficient and major contributors to the inhibition of the corrosion of mild steel in industrial wastewater.

Vegetable peels, leaves, and fruits are rich in phytochemicals (including alkaloids, saponins, glycosides, and tannins) [48]. The leaves of the plants also contain certain beneficial nutrients such as mineral elements and secondary metabolites, which explain their medicinal status and possible potency for several pharmaceutical applications [49,50]. Because of their phytochemical contents, some investigations have been carried out to explore their usefulness in corrosion prevention, as discussed below.

### 4.1. Gnetum Africana

*Gnetum africana* is a popular vegetable in several countries, including Nigeria, Cameroun, and others. Eddy et al. [51] reported on the corrosion inhibition effect of ethanol extract of *Gnetum africana* for mild steel in a H_2_SO_4_ system. The concentration range employed was 0.1 to 0.5 g/L and the ranges for the evaluated inhibition efficiency were from 43.33 to 72.1% and from 60.53 to 67.11% at 303 and 333 K, respectively. The efficiency of this vegetable was calculated using three different methods (namely gravimetric, gasometric, and thermometric methods) and the results obtained from the three methods using Equations (6)–(8) were consistent with each other and displayed the general trend expected for adsorption inhibitors. The performance of the inhibitor was linked to the adsorptive powers of anthraquinone, saponin, alkaloid, tannin, terpene, cardiac glycoside, and alkaloid from the extract. The adsorption was exothermic (i.e., values of enthalpy change were negative) and spontaneous (the free energy change was negative), while the prevailing mechanism of adsorption was physical adsorption, judging from the observed trends that reflected a decrease in inhibition efficiency with temperature and lower values of standard free energy change (which ranged from 22.42 to 77.38 J/mol at inhibitor concentrations of 0.1 to 0.5 g/L respectively). Their work investigated the kinetics of the corrosion inhibition process through the calculation of the activation energy at different inhibitor concentrations. They observed that the activation energy increased with the increase in concentration, which pointed to increasing adsorption strength with concentration. Two adsorption isotherms were fitted to the adsorption characteristics of the inhibitor, namely the Langmuir isotherm (Equation (11)) and Temkin isotherm (Equation (12)). Positive interactions (deduced from the Temkin interaction parameter), a confirmed the attraction between the inhibitor’s molecules:

(11) $$log\left(\frac{C}{\theta }\right)=logK-logC$$

(12) $$\theta =-\frac{logK}{2a}-\frac{logKC}{2a}$$

Studies conducted by Nnanna et al. [52] on the application of acid (HCl) extract of *Gnetum africana* as a corrosion inhibitor for carbon steel yielded a maximum efficiency of approximately 95%. The estimated heat of adsorption ranged from −1.32 to 2.65 kJ/mol, which was very low and consistent with the exothermic adsorption and mechanism of physical adsorption. However, the evaluated values of the activated ranged from 26 to 199 kJ/mol and aligned with the physisorption mechanism at low concentrations and chemisorption at higher concentrations. This discrepancy is not abnormal, although judging from the experimental data, which showed that the inhibition efficiency of the extract decreases with a rise in the concentration to 2 g/L of the extract, the conclusion can be drawn that the experimental data predict the physisorption mechanism rather than the chemisorption mechanism, as suggested by the estimated values of the activation energy and the changes in standard free energy of adsorption (range = −3.37 to −12.43 kJ/mol). Another point of argument that leaves these results in theoretical contention is the fact that the activation energies were deduced from corrosion rates obtained from measurements at only two temperatures. Ideally, the statistical approximation of theoretical data for experimental data becomes more reliable as the sample size increases towards the population size.

Obiukwu et al. [53] also reported that the aqueous extract of *Gnetum africana* inhibited the corrosion of mild steel in acid media (HCl and H_2_SO_4_), with a maximum inhibition efficiency of up to 95%. Weight loss measurements were employed to investigate the effects of time on the corrosion and corrosion inhibition of mild steel. Although the authors did not carry out an elaborate investigation on the basic factors that influence the corrosion inhibition, such as the kinetics, thermodynamics, and adsorption behavior of the inhibitor, it can be concluded that the leaf extract of *Gnetum africana* has strong inhibition potential for most metals. Additionally, an investigative study weight loss reported by Nkuzinna et al. [54] indicated the inhibition rules of *Gnetum african leaf* extract for the corrosion of copper, including the application of 2^3^ factorial designs. They reported a retention time of 24 h and an inhibitor concentration of 0.003 g/L as the optimum requirements for the best results. However, this conclusion is at odds withstand existing scientific evidence in the field of corrosion studies. The performance of most adsorption corrosion inhibitors (such as the leaf extract of *Gnetum africana*) is known to increase with an increase in concentration because the increased concentration enhances the availability of the inhibitor at the interface through diffusion. Probably the reason for the gap was that very few factors were considered, whereas corrosion inhibition involves interactions between several physical, chemical, and electronic factors.

A probe into the chemical structures and relative concentrations of the phytochemical constituents of *Gnetum africana* leaf (Table 1) should be able to provide information on the expected contribution of each phytochemical toward corrosion inhibition [55]. From the structures, there is certainly a strong correlation between corrosion inhibition and the chemical constituents of *Gnetum africana* leaf. Therefore, the inhibition of the corrosion of metals by the leaf extract of this plant is achieved through a synergistic combination of all useful constituents.

### 4.2. Lasianthera Africana

*Lasianthera africana* is a popular vegetable plant in Nigeria, Cameroon, the Ivory Coast, and several other countries around the world. The involvement of the leaf of this plant in corrosion inhibition has also been documented. Eddy et al. [38] implemented a comprehensive investigation of the classes of phytochemicals in *Lasianthera africana* and applied the ethanol extract of the plant leaf to inhibit the corrosion of mild steel in H_2_SO_4_ at different temperatures. The plant extracted performed as an effective adsorption inhibitor for mild steel corrosion, strongly supporting the assumptions of the Langmuir and Temkin adsorption isotherms, revealing information on the mechanism of physical adsorption, since the changes in adsorption free energy (evaluated from the Langmuir and Temkin constants, range = −11.01 to −16.88 kJ/mol) under standard conditions did not reach −20 kJ/mol. Experimental data also supported the physisorption mechanism because the inhibition efficiency decreased at higher temperatures. The estimated values of the activation energy and the low exothermic heat of adsorption were also listed as evidence that supported the study. Although they carried out detailed studies through the application of thermodynamics, kinetics, and adsorption theories, the authors did not employ electrochemical techniques to elucidate further information, such as the magnitudes of charge transfer resistance, corrosion current, corrosion potential, and Tafel constants. However, it can be stated that since the maximum inhibition efficiency obtained from weight loss (after seven days of immersion) was 94%, the instantaneous inhibition from the extract would certainly be much higher because weight loss methods measure the average inhibition efficiency while others measure the instantaneous inhibition efficiency, which is normally higher than the average inhibition efficiency. Finally, they found that synergism with halides ions can enhance the efficiency of the extract, while the infrared spectrum was informative in providing knowledge of the functional groups that aided the adsorption of the extract. James et al. [56] also investigated the inhibition of the corrosion of mild steel in H_2_SO_4_ using aqueous extracts from three plant species, namely red peanut skin extract, *Lasianthera africana*, and *P. beauv.* mucilage. The obtained maximum inhibition efficiency (99.49%) was comparable to the value reported by Eddy et al. [38] and showed a proportional relationship with concentration but an inverse relationship with temperature. They also attributed the efficiency of the *Lasianthera africana* leaf to its phytochemical components.

### 4.3. Pumpkin

Okewale et al. [57] provided a brief report on the effectiveness of a pumpkin pod extract as a corrosion inhibitor and provided a statistical relationship between the inhibition efficiency and factors that affect it, such as the time, concentration, and others. A detailed study to elucidate the mechanism of inhibition was not reported in their work. Pumpkin seeds have been employed by Radi et al. [58] to inhibit the corrosion of aluminum in saline solution (NaCl), with a maximum recorded efficiency of 95% at the inhibitor concentration of 1 g/L. Atomic force microscopy, scanning electron microscopy, and X-ray diffraction studies have revealed information on the protection of the surfaces of metals when corrosion was inhibited compared to metals in blank solution. Electrochemical methods employed for such studies indicated that the extracts acted as cathodic inhibitors, while the results of curve fittings to adsorption isotherms indicated the Langmuir isotherm as the best-fitting isotherm. Nwachukwu et al. [44] adopted different blends of guava and pumpkin leaves to inhibit the corrosion of mild steel in hydrochloric acid and found an optimum inhibition efficiency of 95%. They reported that the combined extracts contained various phytochemicals and that the inhibition efficiency was dependent on the period of contact, the concentration of the inhibitor, and the temperature. Based on the application of response surface analysis, the authors concluded that the experimental inhibition efficiency was very close to the proposed theoretical value. However, due to the openness of the corrosion inhibition mechanism, this projection may fail. The work was based on weight loss only and did not use other established methods of analysis. The data sample size might not have been close to the expected population data, in addition to other interactions. Despite this, it is evident from the results obtained that the leaves of this plant were a major contributor to the corrosion inhibition process. Anaee [59] reported good inhibition efficiency (range = 72.4 to 75.7%) against the corrosion of carbon steel in a petroleum environment between 323 and 353 K. The results were obtained using different investigative methods, namely galvanostatic polarization, open circuit potential measurement, optical microscopy, and Fourier transform infrared spectroscopy techniques. Increases in the quantity of the extract led to increased efficiency. Potentiodynamic polarization plots indicated the inhibitor to be a mixed-type inhibitor. The adsorption fitted the Langmuir isotherm and was consistent with the physisorption mode. Mailbulangu et al. [60] also acknowledged the inhibitory role of pumpkin leaf toward the corrosion of copper in an acidic medium. They observed characteristics that are typical of adsorption inhibitors with physisorption mechanisms.

The above review showed that the extract’s inhibition efficiency is certainly a function of the phytochemical constituents of the leaf. This information is contained in Table 2 [55], which shows that most components of the leaf extract meet the structural requirements concerning corrosion activity.

### 4.4. Ocimum Gratissimum

*Ocimum gratissimum* leaf is a common spice found in many traditional foods and is also used as a vegetable in most countries. The plant is widely distributed across Africa, Mexico, Bolivia, Southern Asia, Brazil, and other countries. Several reports have documented the application of the leaf extract of *Ocimum gratissimum* for the inhibition of certain metals. Adindu et al. [61] adopted certain investigation tools (gravimetric, electrochemical, FTIR, and scanning electron microscopy) to investigate the inhibition of the acidic corrosion (0.5 M H_2_SO_4_ and 1 M HCl) of mild steel by *Ocimum gratissimum* leaf extract. They obtained inhibition efficiency values of 92.4 and 71.1% for the metal in HCl and H_2_SO_4_, respectively. From the FTIR spectra of the inhibitor and corrosion products, the functional groups responsible for the adsorption of the inhibitor were deduced, while the scanning electron micrograph revealed the smooth surfaces of the metals, the corrosion of which was inhibited. Alinnor [62] also observed that aqueous extract (79.79 to 95.65%) of *Ocimum gratissimum* leaf inhibited the corrosion of aluminum in an acidic medium more so than ethanol (68.62–92.74%) and acid (HCl) extract (55.96 to 90.72%). Langmuir and Florry–Huggins isotherms (Equation (13)) supported their work:

(13) $$log\left(\frac{\theta }{C}\right)=logk_{FH}-\log\left(1-\theta \right)$$

Estimated changes in free energy suggested a physical adsorption process to be responsible, while the heat shown in the adsorption data indicated that the adsorption was exothermic. Shreds of evidence gathered from the study conducted by Madawa et al. [63] indicated that an extract of *Ocimum gratissimum* leaf performed better than cassava and neem as an inhibitor for a pipeline in a sodium sulphate environment. Sharma et al. [64] also showed that the leaf extract of a similar species to *Ocimum gratissimum, Ocimum tenuiflorum,* showed maximum inhibition efficiency values of 82.25 and 70.15% at 303 and 333 K, respectively, for the corrosion of zinc in H_2_SO_4_ solution Similarly, Solomon et al. [65] showed through weight loss measurements that the leaf extract of *Ocimum gratissimum* inhibited the corrosion of stainless steel in a biologically active environment. Perhaps the most extensive documented explanation for the inhibition of *Ocimum gratissimum* leaf extract is the one reported by Eddy et al. [66] for mild steel in H_2_SO_4_ solution. They used thermodynamic, kinetic, and adsorption theories to explain the inhibition behavior of the extract. FTIR was used to identify the functional group associated with the adsorption process. The adsorption of the extract was of the Langmuir type, in addition to being exothermic and spontaneous, and was attributed to the physisorption mechanism due to the observed decrease in inhibition efficiency at higher temperatures and the magnitudes of the estimated changes in standard Gibbs free and activation energies.

### 4.5. Murraya Koenegii

Beenakumari [67] successfully inhibited the corrosion of mild steel in 1 M HCl using a leaf extract of *Murraya koenigii* with weight loss, polarization (potentiodynamic), and open circuit potential measurements. He observed a maximum inhibition efficiency of 94.2% (at an extract concentration of 1000 ppm) after 120 min of immersion (i.e., from weight loss measurements). The inhibition effect improved with an increase in the period of contact and at lower temperatures. Harmonious results were observed in the weight loss and electrochemical data. Relatively similar results were observed by Sharmila et al. [68] for the inhibition of carbon steel in HCl solution. The inhibition efficiency ranges were from 80.4 to 84.6%, 78.6 to 82.4%, 81.2 to 84.5%, and 81.3 to 84.6% for data obtained from gravimetric, gasometric, polarization, and impedance spectroscopy measurements, respectively. The inhibitor was more anodic than cathodic and its behavior aligned with the Langmuir adsorption model. The report published by Pushpanjali et al. [69] also confirmed the leaf extract of *Murraya koenigii* as an anodic inhibitor of the corrosion of aluminum in HCl solution. They obtained an optimum inhibition efficiency of about 92% at a very low extract concentration (92%). The adsorption of the extract proved the Langmuir model to act through the combination of physisorption and chemisorption mechanisms. Additionally, Yadav et al. [70] reported on the successful implementation of *Murraya koenigii* leaf extract for the inhibition of the corrosion of mild steel in a 0.1 N trioxonitrate (V) acid environment. The study indicated an inhibition efficiency of 62%. The recorded parameters were the activation energy (18 kJ/mol), enthalpy change (15.46 kJ/mol), entropy change (−251 J/mol), and free energy change (−28.28 kJ/mol), which led to the conclusion that the adsorption of the inhibitor that best-fitted the Temkin adsorption model was ordered, endothermic, and spontaneous and gave evidence for a physisorption mechanism.

Chukwueze et al. [28] found that curry, neem, and pawpaw leaves contain high proportions of tannin that gave them very good corrosion inhibition effects for mild steel in sulphuric acid. The investigated factors that influenced the performance of the inhibitor were the temperature, contact time, and concentration. The scanning electron micrograph of the metal surface confirmed the effectiveness of the extract in the inhibition of the investigated metal corrosion. An in-depth investigation employing both theoretical and experimental tools toward the efficient inhibition of mild steel corrosion in 5 M HCl was carried out by Kumar and Yadav [71] to obtain a maximum inhibition efficiency of 98.13% at an inhibitor concentration of 0.5 g/L in 5 M HCl solution. Their study revealed the inhibition through physico-chemisorption based on the magnitudes of the adsorption energy, interaction energy, and parameters deduced from Frumkin and Langmuir isotherms. A maximum inhibition efficiency of 98.5 was obtained for mild steel in CO_2_-saturated 3.5% saline (NaCl) solution by Amir et al. [33]. The involvement of functional groups in adsorption (FTIR analysis) and the smoothness of the surface in the presence of leaf extract of *Murraya koenigii* provided evidence to support the information obtained from polarization (potentiodynamic) and mass loss methods. Other successful reports on the application of leaf extract for the inhibition of the corrosion of metals are those published by Umarani et al. [72] for cast iron in drinking water and harmless tertiary inhibitors. The GCMS spectrum of curry leaf extract contains alpha.-caryophyllene, benzene, 1-dimethylamino-4(2-cyano-2-phenylethenyl, 2-phenyl-4quinolinecarboxamide, phenanthrene, 9,10-diethyl3,6-dimethoxy, 10H-phenoxaphosphine, 2chloro-8-ethyl-10-hydroxy, and 1,5-diformyl-2,6-dimethoxyanthracene [73]. These compounds meet all of the requirements for organic corrosion inhibitors. Therefore, the excellent inhibition efficiencies reported in this review for the leaf extract in various environments and various metals are also attributed to the phytochemical constituents. In Table 3, we present a list of other vegetables that have been applied as corrosion inhibitors for metals.

## 5. Fruit Juices as Green Corrosion Inhibitors

Several fruits juices are effective in protecting most metals against corrosion. Oladele and Okoro [83] reported low inhibition efficiencies (up to a maximum of 40%) for the corrosion protection of aluminum in H_2_SO_4_. The low efficiency may have been due to the passivating nature of aluminum, which has the potential to form a protective coating on itself. Therefore, although the corrosion is not rapid, inhibition is expected to be below. Additionally, the juice was used in its natural form, without concentrating its components. The major chemical compound in orange juice is ascorbic acid, which is known to be a good corrosion inhibitor. For example, in 3.5% NaCl solution, the inhibition efficiency of ascorbic acid against carbon steel was reported to be 95.4% by Irwan et al. [84] and 88.96% for reinforced concrete in a saline environment. Therefore, orange has constituents that are good corrosion inhibitors. Sikachina [85] also reported fresh juice from *Citrus recticulata, Solanum lycopersicu, Viburnum opulu, Vitis vinifera,* and *Malus domestica* as effective corrosion inhibitors for aluminum in an alkaline medium. Statistical tools were employed to trace the relationships between the contribution factors, such as the concentration and chemical constituents. However, limited information was provided by the authors in their corrosion research. The results of the investigation of the inhibitory role of apricot fruit juice on the corrosion of mild steel in phosphoric acid by Yaro et al. [86] indicated good inhibition efficiency. The Langmuir isotherm was most appropriate for the adsorption of the inhibitor, and the efficiency of the inhibitor was found to be a function of the concentration, temperature, and degree of interaction. The evaluated activation energy levels (which ranged from 42.02 to 45.07 kJ/mol) were within the boundary that defines the mechanism of physical adsorption. The enthalpy changes in all cases equaled approximately 40 kJ/mol and were positive, suggesting an endothermic adsorption process. However, the low and negative values of entropy changes are indicative of the ordered adsorption process. The inhibition efficiency levels of the apricot juice varied from 63 to 72% between concentrations of 10 and 40 g/L.

Yaro et al. [87] also established that peach fruit extract is a good corrosion inhibitor for mild steel in HCl solution. A maximum inhibition efficiency of 91% was recorded at 323 K. The mechanism of physical adsorption was sustained for the adsorption of the extract. The adsorption also showed strong obedience to the Langmuir model. Juice from *Prunus cerasus* has also been documented to be an active corrosion inhibitor for steel in HCl solution [88]. Polarization measurements gave very high inhibition efficiency levels (i.e., 89.5 to 94.1%) for extract concentrations in the range of 0.3 to 4.0 v/v%, while 85.17 to 92.69% was the range for the inhibition efficiency levels measured using the electrochemical impedance spectroscopy method. The corrosion inhibition efficiency decreased for every increment of temperature and gave evidence that the adsorption of the inhibitor supported the mechanism of physical adsorption. Apple juice has also been confirmed to be an efficient inhibitor of the corrosion of steel in acidic medium [10]. The inhibition was attributed to the presence of 1-linoleoyl-sn-glycero-3-phosphocholine in the apple extract. The quantum chemical calculation gave sufficient information on the model and site of adsorption of the inhibitor. Although thermodynamics and kinetics investigations were not implemented in their study, the provided evidence sufficiently confirmed that apple juice is a good corrosion inhibitor for the studied system and environment.

Tomato juice was found to be very efficient in inhibiting the corrosion of brass and stainless steel but accelerated the corrosion of galvanized steel [89]. The pH of the juice decreased in acidity after thirty days of immersion of brass and stainless steel but changed to neutral after five days and to alkaline after thirty days in solution containing galvanized steel. This suggests severe interactions between the galvanized steel and tomato juice. The inhibition process was monitored through weight loss and polarization methods. The ability of fruit juices from tomato (pH = 4.24), pineapple (pH = 3.94), orange (pH = 3.58), and lemon (pH = 2.22) to catalyze the corrosion of carbon steel was observed to be highly dependent on pH. The more acidic juice from lemon resulted in the highest corrosion rate (2.89 mm/year), while the metal in the least acidic tomato juice had the lowest corrosion rate (0.86 mm/year). The corrosion rates for the metals in pineapple and orange juices were 1.81 and 1.52 mm/year, respectively [90,91]. In the acidic environment (HCl), the corrosion rate was also dependent on the pH. At HCl concentrations of 0.01, 0.001, 0.0001, and 0.00001 M, the corrosion rates were 2.19, 0.38, 0.17, and 0.04 mm/year, respectively. Consequently, at low pH values the juices catalyzed the corrosion, while at higher pH the corrosion rate was retarded, which suggests that these juices are better corrosion inhibitors in alkaline media than in acidic media. The results from their work also suggest that carbon steel containers are not suitable for the storage of these fruit juices. Saeed et al. [7] invested the effects of carrot juice on the corrosion of mild steel in HCl solution. They reported that the corrosion rate of the metal (in a test solution containing the juice) increased with temperature. However, a progressive increase in the concentration of the carrot juice manifested in a reduced corrosion rate. Regarding the chemical constituents of the carrots, the strong inhibition potential is certainly due to the presence of carotene. The study of the corrosion of metals in the presence of fruit juices is very significant because of their dual roles (i.e., acceleration or inhibition of corrosion).

## 6. Conclusion and Future Prospective

In this study, typical applications of fruits and vegetables have been reviewed regarding corrosion inhibition. The basic requirement for any material to be a good corrosion inhibitor is the possession of structural factors such as heteroatoms, aromatic systems, conjugated systems, and multiple bonds. This requirement is easily satisfied by most of the phytochemicals in fruits and vegetables. Vegetables and fruits extracts have the outstanding advantages of being natural, biodegradable, and non-toxic, meaning fruits and vegetables are highly popular products for corrosion inhibition applications. Due to their high demand in the corrosion protection sector, this may cause future food insecurity due to the continuous use of food materials for non-food purposes.

## Figures and Tables

**Table 1:** Chemical constituents of *Gnetum africana* leaf.

Compound	%C	Chemical Structure
2-Cyclopenten-1-one, 2-hydroxy-3,4-dimethyl-	8.59	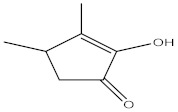
Estragole	6.45	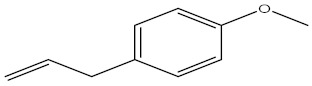
2-methoxy-4-vinylphenol	10.94	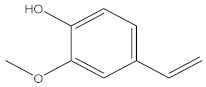
5-chloropentanoic acid 4-methylpentyl ester	1.76	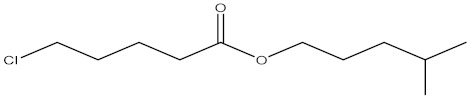
1,3 Benzodioxole, 4-methoxy-6-(2-propenyl)	3.32	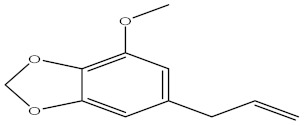
Tetradecanoic acid	9.77	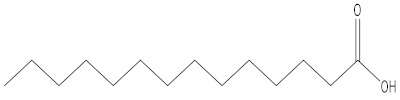
Hexyl amine	3.32	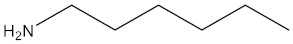
Caffeine	18.95	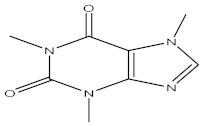
3,4,6-Tri-O-methyl-D-glucose	2.34	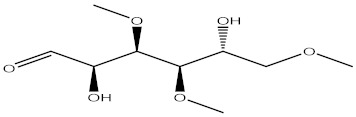
Palmitic acid	11.91	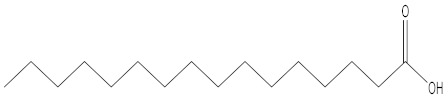
phytol	7.62	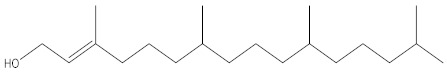
Linoelaidic acid	3.91	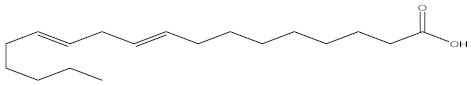
Cyclopentaneundecanoic acid	9.38	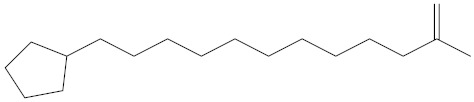
Cis, cis, cis-7-10-13-Hexadecatrienal	1.76	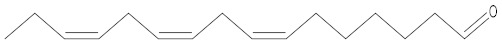

**Table 2:** Some important phytochemicals in pumpkin leaf extract.

Name of Compound	Chemical Structure
n-Amylcyclohexane	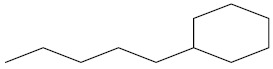
L-Proline, 5-oxo-, methyl ester	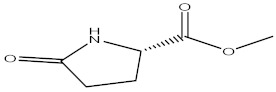
Hexahydroindole	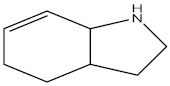
2,4-Imidazolidinedione, 5-methyl	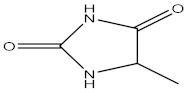
9,12,15- Octadecatrienal	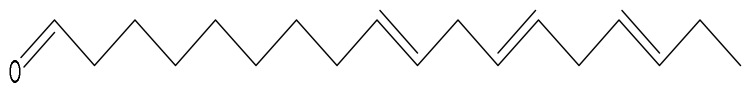
6- Octen-1-ol, 3,7-dimethyl (±)	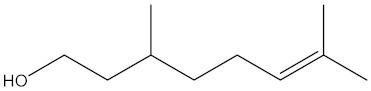
n-Hexadecanoic acid	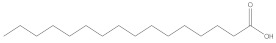
E,E-1,9,17- Docasatriene	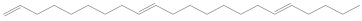
3- [Prop-2-enoyloxy]tetradecane	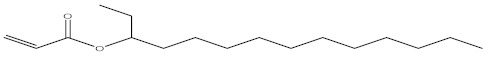

**Table 3:** Some vegetables reportedly used as corrosion inhibitors.

Other Plants (Leaf)	Metal and Medium	IE (%)	Mechanism	References
*Gongronema latifolium* leaf	Steel in H_2_SO_4_ and HCl	90.95		[53]
	H_2_SO_4_	90.12	Physisorption	[74]
	Al in KOH and HCl	97.54	Chemisorption	[75]
	Steel n HCl	91.69	Physisorption	[76]
*Piper guinensis*	Steel in H_2_SO_4_	94.00	Physisorption	[77]
*Vernonia amygdalina*	Steel in H_2_SO_4_	82.89	Physisorption	[78]
	Steel rebar in H_2_SO_4_	60.68	-	[79]
	Steel in 3.5% NaCl	75.00	Physisorption	[80]
	Steel in H_2_SO_4_	87.00	Physisorption	[81]
*Colocasia esculenta*	Steel in HCl	98.43	Physisorption	[82]
*Solanum melongena*	Steel in H_2_SO_4_	82.21	Physisorption	[13]

## Data Availability

Not applicable.
